# Molecular pathology in breast disease: diagnostic, prognostic, and therapeutic tools

**DOI:** 10.1007/s00428-023-03709-0

**Published:** 2023-11-28

**Authors:** Zsuzsanna Varga, Umberto Maccio

**Affiliations:** Department of Pathology and Molecular Pathology, University Hospital Zurich, Schmelzbergstrasse 12, CH-8091 Zurich, Switzerland

**Keywords:** Breast cancer, Carcinomas, Molecular testing

## Abstract

Molecular testing in breast cancer gained increasing attention and importance as specific molecular results can tailor not only oncological decisions on systemic adjuvant or neoadjuvant or in metastatic setting, but increasingly serve in diagnostic routine histopathological services to differentiate between morphologically overlapping or ambiguous histological pictures. Diagnostic tools involve in most cases a broad spectrum of immunohistochemical panels, followed by entity-specific in situ hybridization probes and in given cases NGS-based sequencing. Workflow of which methodology is applied and in which order depends on the specific entity resp. on the given differential diagnosis in question. Regarding prognostic/predictive molecular testing, the choice of assay and the workflow are based on clinical algorithms and on the evidence of targeted therapies following the molecular alterations. In this review paper, we aim to address the use of molecular technics in [1] the histological diagnostic setting (such as subtyping of invasive carcinomas/malignant spindle cell tumors and sarcomas and some B3 lesions) and [2] in the context of adjuvant or neoadjuvant or other clinical settings with special focus of targeted therapies.

## Molecular assays in histological diagnoses

Diagnostic molecular tools additionally to the broad use of immunohistochemistry (IHC) became an important possibility in the differentiation of several entities in breast pathology. These ancillary technologies encompass a wide range of in situ hybridization (ISH) assays as well as sequencing technologies (such as NGS, next generation sequencing) to search for the evidence of specific molecular alterations. ISH assays detect gene amplification, translocations, aberrant signals, allelic loss and fusions, while NGS panels are mostly applied in order to detect specific gene variants (e.g., pathogenic single nucleotide variants, nucleotide deletions and/or insertions, duplications); however, NGS-based tests can also quantify tumor mutational burden (TMB) and detect allelic loss, microsatellite instability and gene amplification. Both ISH and NGS are important tools additionally to conventional morphology and immunohistochemical staining panels, which have been increasingly used in the proper histological typing of several entities in breast pathology as elucidated below. The first part of the paper deals with use of molecular techniques in subtyping of invasive carcinomas/malignant spindle cell tumors and sarcomas and the some B3/B2 lesions.

## Subtyping of invasive breast carcinomas with molecular testing

Several subsets of invasive breast carcinomas may undergo molecular testing with the primary aim of a correct histological diagnosis. These tumors encompass a large group of histologically basaloid appearing carcinomas including triple negative (TN) NST (non special type) carcinoma but also other subtypes such as lobular or luminal type NST carcinomas.

**Fig. 1:**
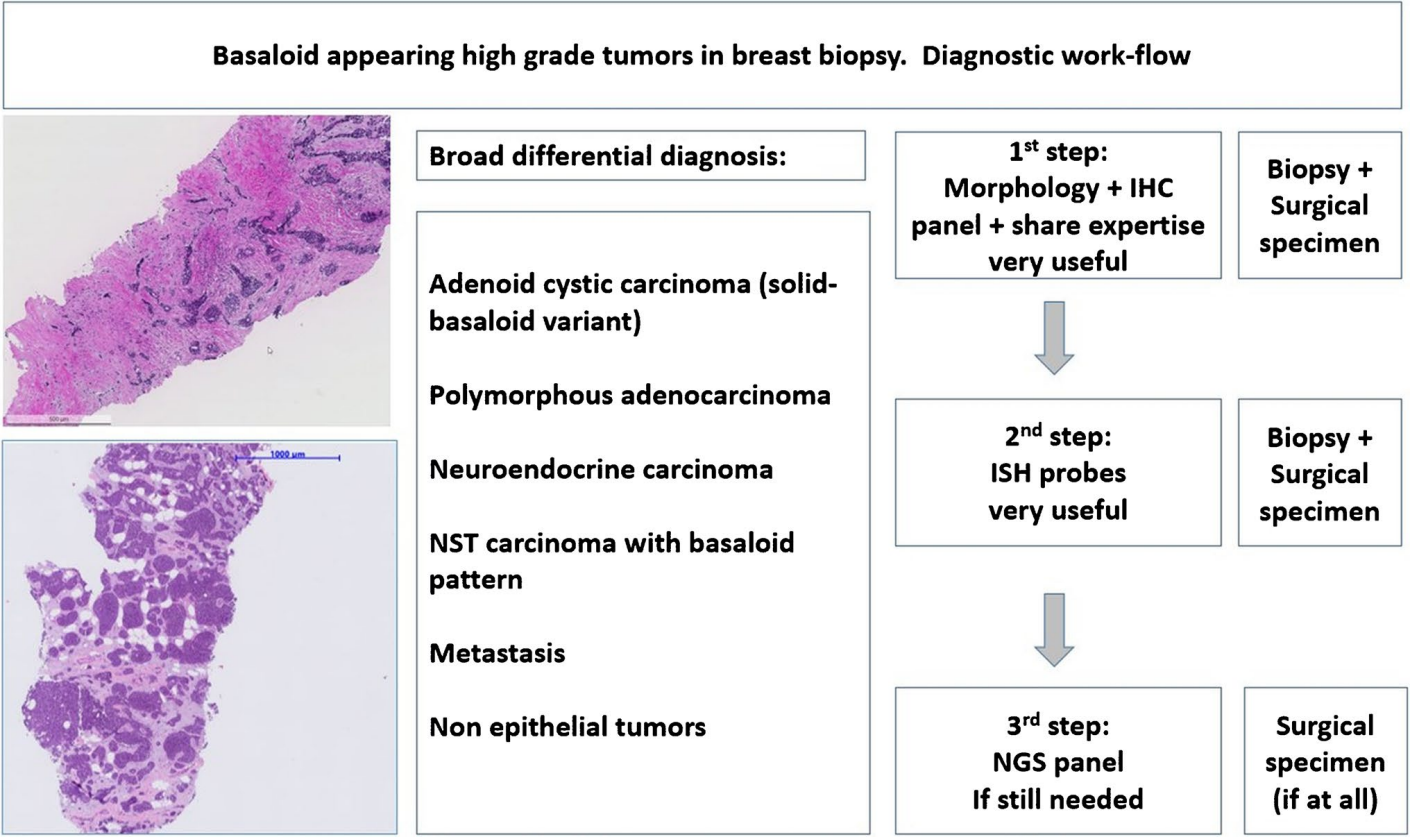
Basaloid appearing high-grade tumors in breast biopsy. Diagnostic work-flow

In case of basaloid appearing carcinomas, the first step in the diagnosis of all these entities is morphology accompanied by a broad panel of immunohistochemistry, followed by in situ hybridization if a definitive diagnosis is still not possible on hematoxylin and eosin (HE) and IHC stains. As last step, NGS assays can be performed if necessary. However, the decision to apply ISH and/or NGS on a core needle biopsy first or to postpone these tests (especially NGS) on the surgical specimen should be met in light of the possible morphological differential diagnoses and after having discussed the next therapeutic decisions in a multidisciplinary meeting (MDM). In such cases, decicions should preferentially include also experts in surgery and oncology of soft tissue and head and neck tumors (Fig. 1, Table 1).

**Table 1:** Spectrum of basaloid appearing tumors in the breast. Characteristic immunohistochemical stains and molecular tests

Diagnosis	HE	ER/PR/Her2	CD117	CK7	BCL2	SOX10	CK5/6	p63/p40	Neuroendocrine markers	COLL IV	ISH aberration	Gene alteration	Breast-specific markers
AdCC SB variant	Basaloid	TN	+++ luminal	+++ luminal	Variable +/−	+++	+++ luminal/basal	++ basal	Absent	**+++ pseudoluminal**	**MYB** **MYBL1**	**MYB** **MYBL** CREBBP NOTCH1 KMT2C	Variable +/++
Polymorphous carcinoma	Basaloid	TN	+++	**Weak or absent**	**+++**	+++	+++	Negative	Absent	Absent	Not available	SF1R, ERBB3, FGFR2, HNF1A, JAK2, **PRKDR1,** SMO, STK11, TP53	++
Neuroendocrine carcinoma	Basaloid	**Luminal (40%)**	Variable +/++	Variable +/++	Variable +/++	++ (in TN)	+/++	Absent	**+++ (diffuse)**	Absent	Not relevant	TP53, PIK3CA RB1	Variable ++/+++
NST with basaloid pattern	Basaloid	TN or luminal	Variable +/++	+++	++	+++ in TN	Variable +/+++ in TN	Variable +/+++ in TN	Absent or focal +	Absent	Not relevant	BRCA1/2 PIK3CA p53 ERBB2 CDH1 PTEN	Variable ++/+++
Metastases	Basaloid	Luminal or TN	Depends on the primary tumor										**Absent**

**Bold** indicates diagnostic entity specific tests. *SB*, solid basaloid; *ER*, estrogen receptor; *PR*, progesterone receptor; *TN*, triple negative

### High-grade adenoid cystic carcinomas (solid-basaloid and high-grade transformed variant)

This entity presents as basal appearing tumor with triple-negative (ER/PR are negative and *ERBB2*/Her2 is not amplified) phenotype and established immune expression [1]. If the immunohistochemical panel (details in Table 1) is not conclusive, FISH break apart probe for detecting rearrangements of the *MYB* gene should be performed. A rearrangement (typically in form of a split signal) is diagnostic, since it is indirectly indicative of the *MYB::NFIB* fusion t(6;9)(q22–23;p23–24). More rarely, an amplification of the MYB or a loss of 3′ part in the break apart FISH, indicative of an unbalanced translocation is present. All these described alterations lead to overexpression of the MYB protein, which can be detected with immunohistochemistry [2].

Advantages of the FISH assay are the sensitivity and specificity and the short turnaround time, which allows a rapid diagnosis. However, though not required for the diagnosis, the fusion partner remains unknown,  although this can be detected through a RNA-based NGS-analysis. If the FISH analysis for MYB is negative, a second FISH for MYBL1 can be performed. In fact, rearrangements of MYBL1 account for the majority of AdCCs without the classical *MYB::NFIB* translocation [3]. These cases show typically two main fusions (*MYBL1::ACTN1* and *MYBL1::NFIB*), both leading to MYBL1 overexpression. Alternatively, if the MYB FISH is negative, an NGS-analysis can be considered as next step. It is of note, the ISH/NGS tests on MYB/MYBL1 genes have a high detection level, especially in classical type of AdCC and are less sensitive in high-grade variants. In such instances, further genes, such as NOTCH1, RAS, and SMARCA5, can be examined with NGS panels including those genes in their pipeline [1, 4].

### **Polymorphous adenocarcinoma**

This rare subtype, which has been only described in isolated case reports, represents another triple-negative carcinoma with basaloid morphology and a characteristic immunohistochemical profile [1]. It is of note, the only one such case of the breast undergoing NGS analyses showed alterations in several genes such as SF1R, ERBB3, FGFR2, HNF1A, JAK2, KDR, SMO, STK11, and TP53, even though these mutations are not specific for the diagnosis of mammary polymorphic adenocarcinoma [5]. Interestingly, this entity in head and neck regions has been shown to have PRKD1 mutations in de novo cases and PRKD2 rearrangements in recurring cases. Nevertheless, such association has not been shown in mammary cases [6].

### **Neuroendocrine tumors/carcinoma**

Neuroendocrine tumors/carcinomas often represent a luminal phenotype with high ER/PR expression and with diffuse expression of neuroendocrine markers. Molecular alterations, such as PIK3CA mutations in low-grade neuroendocrine tumors, can be detected, whereas TP53 and RB1 alterations with loss of functions are typically found in neuroendocrine carcinomas. However, these mutations are not specific for the diagnosis of neuroendocrine breast tumors as these mutations can occur in higher frequency in other tumor entities, such as NST carcinomas as well [1].

### **NST (non-special type) carcinoma with or without basaloid pattern**

According to the 2019 WHO classification of breast tumors, basaloid differentiation within an otherwise NST carcinoma is no longer classified as separate entity, but is considered as a subset of NST carcinomas and is further classified into intrinsic groups based on ER/PR/Her2 expression such as luminal, TN, and Her2-positive subgroups [1, 7]. Underlying molecular changes, though numerous, are not diagnostic for an NST carcinoma and are not considered in tailoring first line clinical therapy decisions.

- *TN phenotype*: The TP53 gene has the highest frequency of mutations in this group. TN NST carcinomas often harbor mutations in BRCA1/2, PTEN, and RB1 genes as well.
- *Luminal phenotype*: The PIK3CA gene is the most frequently mutated gene (up to 40%). Further mutations in luminal NST carcinomas involve genes such as ESR1, (especially a progression mutation after hormonal therapy), FGFR1, MDM4, AKT1, and GATA3 [1, 7].
- *Her2 phenotype*: ERBB2 gene amplification leading to Her2 overexpression occurs in 10–15% of breast cancer, the majority of which are NST carcinomas. ERBB2 activating mutations, on the other hand, are often detected in breast carcinomas lacking ERBB2 amplification and may be found also in other histological subtypes, such as lobular carcinoma [1, 8, 9].

In the primary therapeutic setting, the expression of ER/PR and Her2 status, as well as proliferation index (Ki67), tailor the decision towards systemic therapy [1, 10–12].

### **Lobular breast carcinoma**

This subtype is mostly luminal type exhibiting common genetic alterations such as gains and losses of chromosome 16q/16p, which are located on the CDH-1 gene, also resulting in E-Cadherin protein loss in >80% of invasive lobular carcinomas [1, 10–12]. Furthermore, PIK3CA mutations are common in up to 40–50% of lobular carcinomas [1, 10–12]. Differently from NST carcinomas, lobular tumors more often exhibit mutations in ARID1A, PTEN, FOXA1, and Her2 genes and less frequently in GATA3 and MAP2K4 genes [1].

### **Metastatic tumors into the breast with basaloid morphology**

Metastatic tumors into the breast are challenging, as in absence of known clinical history and/or of in situ components, extra-mammary tumors can mimic mammary primary, leading to false tumor classifications and suboptimal therapeutical approaches. The use of a broad immunohistochemical panel of breast-specific markers (GATA3, CK7, SOX10, GCDFP-15, Mammaglobin, NY-BR-1) weighted against markers suggestive of other tumor entities (e.g., PAX8, MelanA, TTF1, RCC) can secure the diagnosis of a breast primary and rule out a metastatic lesion. Detection of genetic alteration in diagnostic setting is of limited use, as the mutations are generally not specific of one entity. However, a molecular comparison through a bright NGS panel between a new breast lesion and a known cancer elsewhere can help to demonstrate a clonal relationship and as such framing the lesion in the breast as metastasis [1, 13].

## Molecular testing in salivary-gland type and rare breast tumors

**Fig. 2:**
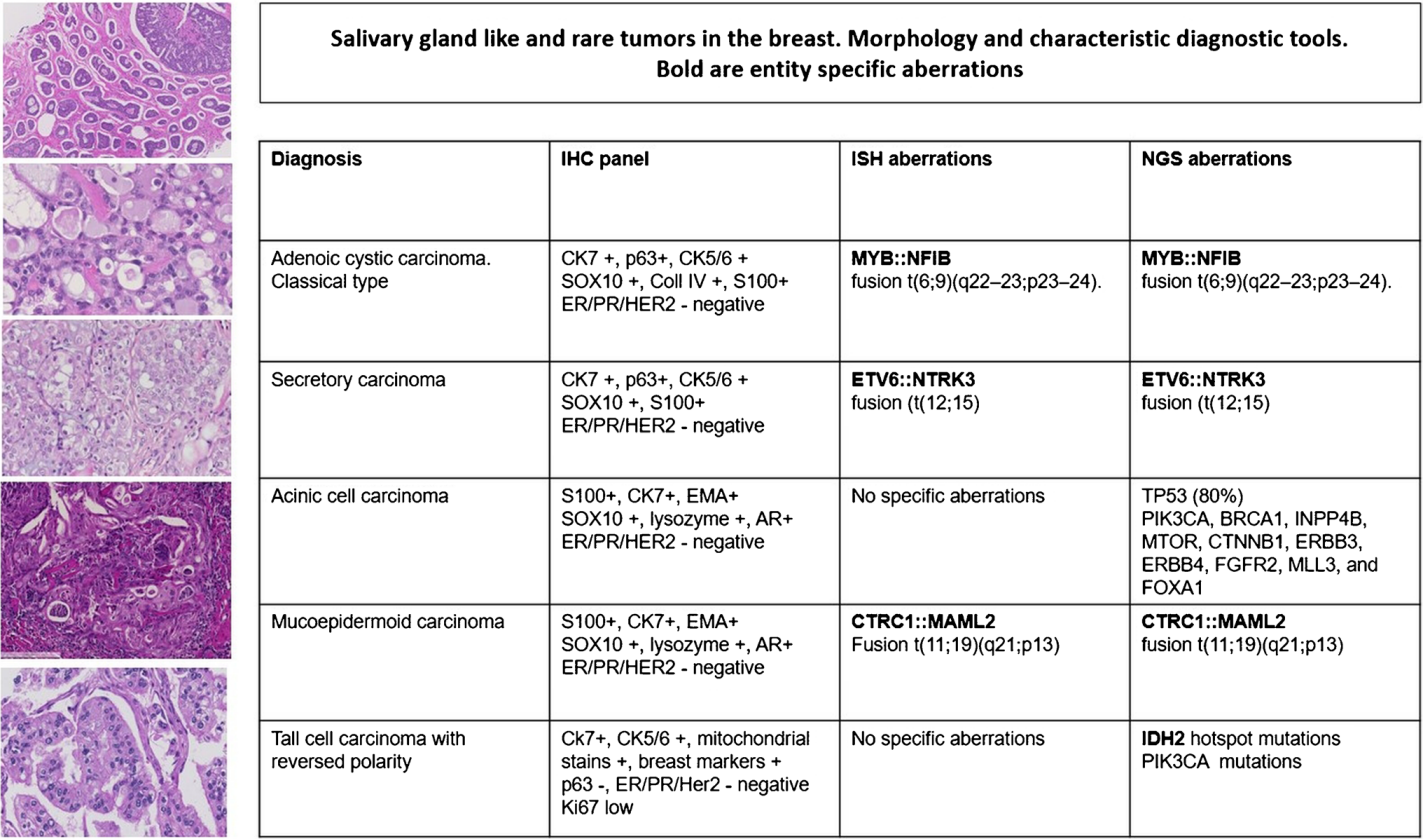
Salivary gland like and rare tumors in the breast. Morphology and characteristic diagnostic tools

Salivary gland type tumors and rare entities in breast pathology pose increasing diagnostic challenges as these tumor types, if not readily recognizable morphologically on HE and with the use of standard immunohistochemical panels, require FISH to demonstrate diagnostic gene rearrangements and/or translocations. Salivary gland like tumors such as adenoid cystic carcinoma (AdCC, see above), secretory carcinoma, mucoepidermoid carcinoma (MEC), polymorphous adenocarcinoma (see above), acinic cell carcinoma (ACC), and tall cell carcinoma with reverse polarity (TCCRP) belong to this category (Fig. 2).

### **Secretory carcinoma**

This tumor is typically triple negative. If through conventional morphological stains and immunohistochemistry the diagnosis is not possible, the detection of *ETV6::NTRK3* fusion (t(12;15) ETS variant 6 (ETV6)–neurotrophic receptor tyrosine kinase 3 (NTRK3) translocation) through RNA-based NGS analysis or the detection of rearrangement of ETV6 gene through FISH break apart probes is diagnostic, if the morphology supports this diagnosis. Positivity for the pan-TRK antibody can serve as a surrogate marker for the *ETV6::NTRK3* fusion [13]. *ETV6::NTRK3* fusion is nevertheless not unique to secretory breast carcinomas as a broad range of tumors with different etiology (such as infantile fibrosarcoma, cellular mesoblastic nephroma, some acute myeloid and lymphoblastic leukemias, some form of papillary thyroid carcinoma, inflammatory fibroblastic tumors, gastrointestinal stromal tumor) can harbor this fusion as well [13–17].

### **Acinic cell carcinomas (ACC)**

This subtype is also triple negative with established immunphenotype [1, 7, 18]. In the majority of ACC-s, there are mutations in TP53 (80%); however, mutations of PIK3CA, BRCA1, INPP4B, MTOR, CTNNB1, ERBB3, ERBB4, FGFR2, MLL3, and FOXA1 have been also described [1, 19]. There are no specific FISH probes or molecular alterations for the diagnosis of ACC, so that the morphological spectrum along with immunohistochemical expression profile are most relevant diagnostic elements.

### **Mucoepidermoid carcinoma (MEC)**

The profile is mostly of a triple-negative carcinoma, with occasional AR expression [1]. Molecular tests with ISH or NGS can be useful especially if diagnostic concern arises. Mastermind-Like Transcriptional Coactivator 2 (MAML2) or the CREB Regulated Transcription Coactivator 1 (CRTC1) rearrangements are common [20–22]. The translocation t(11;19)(q21;p13) resulting in fusion of *MAML2::CRTC1* is diagnostic for MEC, especially in low grade forms [23].

### **Tall cell carcinoma with reverse polarity (TCCRP)**

This very rare subtype is a low-grade triple-negative carcinoma [1, 24–27]. Molecular alterations via NGS such as IDH2 hotspot mutations (the majority) and missense mutations in PIK3CA (also frequent) and other genes of the PI3K pathway have been identified and support the neoplastic nature of TCCRP [13, 28].

Although the above-described molecular changes have been characterized in TCCRP, the diagnosis should be made on conventional morphology and on the immunophenotype irrespectively the molecular changes.

## Molecular testing in metaplastic carcinomas

Metaplastic carcinomas of the breast have undergone relevant biological differentiation and new categorization in the last decades. Low- and high-grade morphological variants with specific histological definitions and underlying molecular changes have been defined and recommendations for the use of systemic therapies have been also addressed based on the morphological pattern and differentiation grade [19, 29–31]. By definition, for the diagnosis, an invasive carcinoma with atypical squamous, spindle cell, and/or mesenchymal/matrix-producing differentiation is required. Of note, in metaplastic carcinomas without an in situ or a conventional-type mammary carcinoma component, direct evidence of epithelial differentiation by immunohistochemistry (such as expression of high-molecular-weight cytokeratins and/or p63) is needed [1]. Low-grade carcinomas encompass the fibromatosis-like and low-grade adenosquamous forms. High-grade variants include spindle cell, squamous subtypes, and those with heterologous components (Table 2).

**Table 2:** Spectrum of metaplastic carcinomas in the breast. Characteristic diagnostic, immunohistochemical stain, and molecular test

Diagnosis	IHC panel	Molecular alterations via sequencing or NGS
Fibromatosis-like carcinoma	Triple negative **Expression of basal markers (CK/p63/CK5/6**)	Claudin-low phenotype TERT promoter mutations Loss of CDKN2A/B
Low-grade adenosquamous carcinoma	Triple negative **Expression of squamous and basal markers (CK/p63/CK5/6, SOX10)**	PIK3CA recurrent mutations TP53 mutations
Spindle cell metaplastic carcinoma	Triple negative **Heterogeneous expression of squamous and basal markers (CK/p63/Ck5/6, SOX10)** **Negative for CD34, b-catenin**	ARID1A, TP53, TERT, or MED12 promoter mutations, mutations in the PI3K or WNT pathways
Squamous carcinoma	Triple negative **Expression of squamous markers (CK/p63/CK5/6)**	No specific mutations
Metaplastic carcinoma with heterologous elements	Triple negative **Expression of mesenchymal markers and CK/p63/Ck5/6**	No specific mutations

**Bold** indicates diagnostic entity specific tests. *ER*, estrogen receptor; *PR*, progesterone receptor; *TN*, triple negative

### **Fibromatosis-like carcinoma (FLMC)**

This is a low-grade triple-negative spindle-cell tumor arranged in fascicles [29, 30, 32).The immunophenotype, which is in most cases triple negative, includes a strong expression of basal cytokeratins (CK5/6, CK14) and p63. Data on gene expression profiling point to claudin-low phenotype, TERT promoter mutations, and loss of CDKN2A/B [27, 33]. Diagnostic criteria of FLMC should be based on HE and immunohistochemistry, irrespectively on the presence of molecular alterations [1].

### **Low-grade adenosquamous carcinoma (LGASC).**

This is another indolent low-grade metaplastic carcinoma variant, which is composed of intermingled squamous and glandular elements arranged in haphazard pattern [29, 32, 34]. LGASC is most commonly a triple-negative carcinoma showing a gene signature typical of the epithelial-to-mesenchymal transition, which is mirrored by the variable expression of basal markers in immunohistochemistry (e.g., CK5/6, p63, SOX10, EGFR) [35]. Molecular alterations involve recurrent PIK3CA mutations and TP53 wild-type mutations [1, 19]. However, on the diagnostic level, conventional morphology and the expression of basal markers are of most relevance and no molecular alteration alone is diagnostic for an LGASC.

### **Spindle-cell metaplastic carcinoma (SCMC)**

**Fig. 3:**
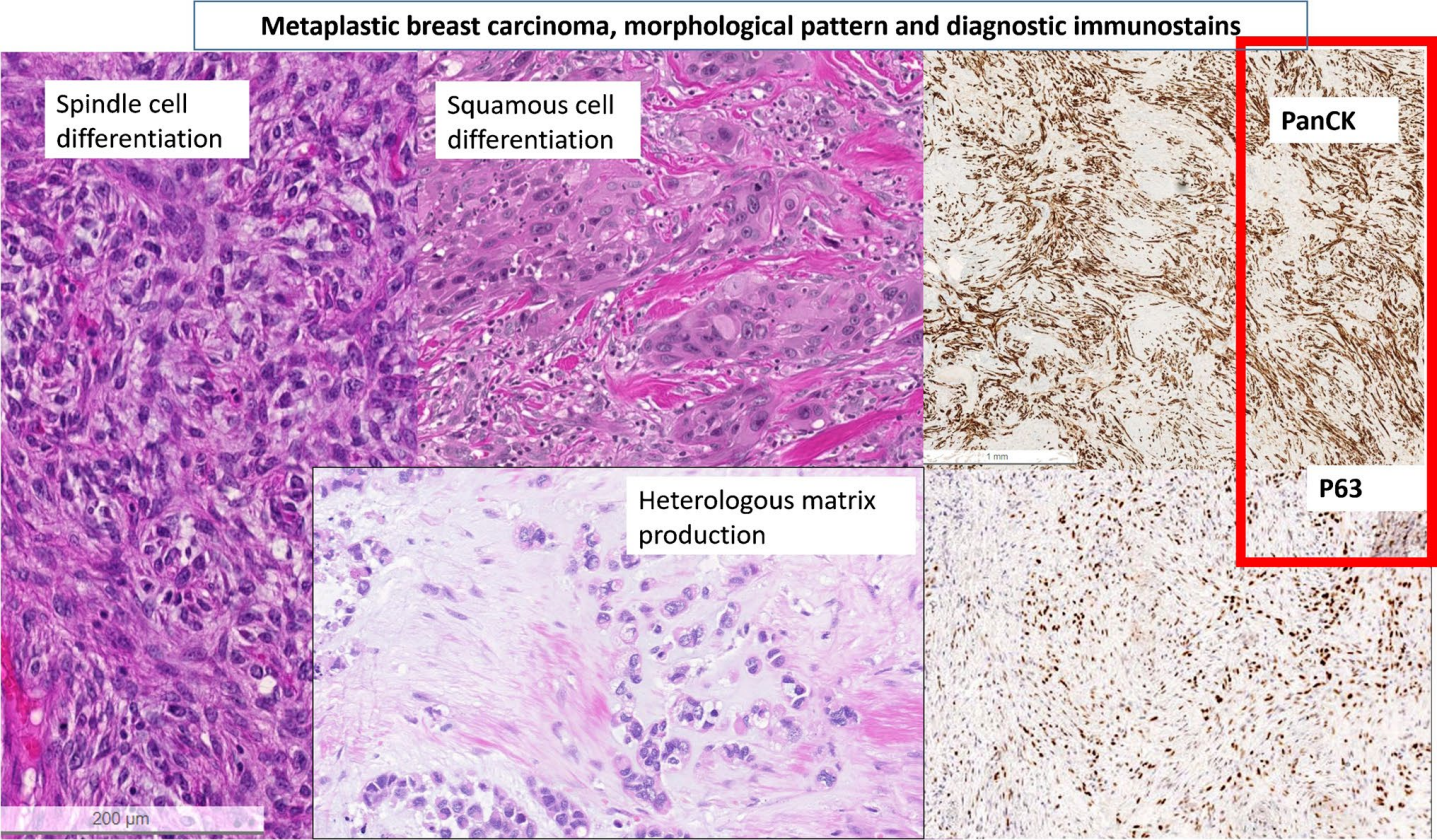
Metaplastic breast carcinoma, morphological pattern, and diagnostic immunostains (PanCK and p63)

Spindle cell carcinoma of the breast is a high-grade form of malignant spindle cell tumors, which is in many instances composed of mesenchymal appearing cells arranged in fascicle-like structures [1, 30, 36]. The main challenge in SCMC is to prove epithelial differentiation and to differentiate it from other malignant spindle cell lesions such as malignant spindle cell stromal component from a phyllodes tumor (PT) or from a sarcoma with spindle cell morphology [1, 30, 36]. According to the last WHO classification, the epithelial differentiation can be extremely challenging to demonstrate, as SCMC often displays a pronounced intra-tumoral heterogeneity, so that the use of immunohistochemical panels including cytokeratin-cocktails and basal markers (such as p63 or CK5/6) to prove SCMC on several tumor areas is oft necessary (as illustrated in Fig. 3) [1]. CD34, β-catenin, and CD10 may be useful in the differential diagnosis with PT [1, 39]. However, CD34 can be reduced or negative, especially in the malignant PTs [1, 37, 38]. Molecular assays, such as NGS-based panels including ARID1A, TP53, TERT, or MED12 promoter mutations, as well as mutations in the PI3K or WNT pathways, aiming to diagnose a sarcoma or a malignant PT can be misleading. In fact, none of the above-mentioned mutations is specific for any of the differential diagnosis listed above (e.g., mutations of TERT promoter may occur in both PT and SCMC). Thus, molecular data should be interpreted within the whole context of the lesion [1]. As a basic rule, in the absence of known history of an extra-mammary spindle-cell tumor, and of typical leaf-like features of malignant PT, especially on core biopsy tissue, caution is required to render any specific diagnosis and to differentiate between SCMC, PT, and sarcoma [1, 30, 36]. The best approach is to sign out a malignant spindle-cell tumor in the absence of any further hints on both HE and immunohistochemistry as such, to categorize the lesion as B5b or B5d and to suggest a primary excision with the aim of a definitive subtyping on the surgical specimen. Increasing data point to reduced response after neoadjuvant chemotherapy in SCMC, PT and sarcoma and endorse a primary excision instead [10, 11, 31, 39]. Consequently, the application of molecular assays is of limited value for the diagnosis of any of these alternatives, and for the definitive diagnosis is best to re-evaluate morphology on the surgical specimen under consideration of the interdisciplinary discussions at the MDM.

### **Squamous cell carcinoma (SMC) and metaplastic carcinoma with heterologous components**

These metaplastic carcinoma forms usually do not pose diagnostic difficulties as the conventional morphology such as recognizable metaplastic components render a straight forward diagnosis also on preoperative core biopsies [1, 30]. These tumor-types are virtually all triple negative and are subjected to treatment guidelines according to metaplastic subtypes and triple-negative intrinsic subtypes [10, 11, 39]. The use of molecular tests is not necessary in most of these cases in the diagnostic setting.

## Molecular testing in B2 (benign) and B3 (lesions of uncertain malignant potential) lesions

In the epithelial types of B3 lesions (such as atypical ductal hyperplasia, flat epithelial atypia, intraductal papillomas, radial scar, classical lobular neoplasia), the diagnosis is solely made on conventional morphology and on corresponding immunohistochemistry (such ER, CK5/6, p63, E-Cadherin/catenin p120 complex) and molecular testing is not useful in any of these entities [12, 40, 41]

**Fig. 4:**
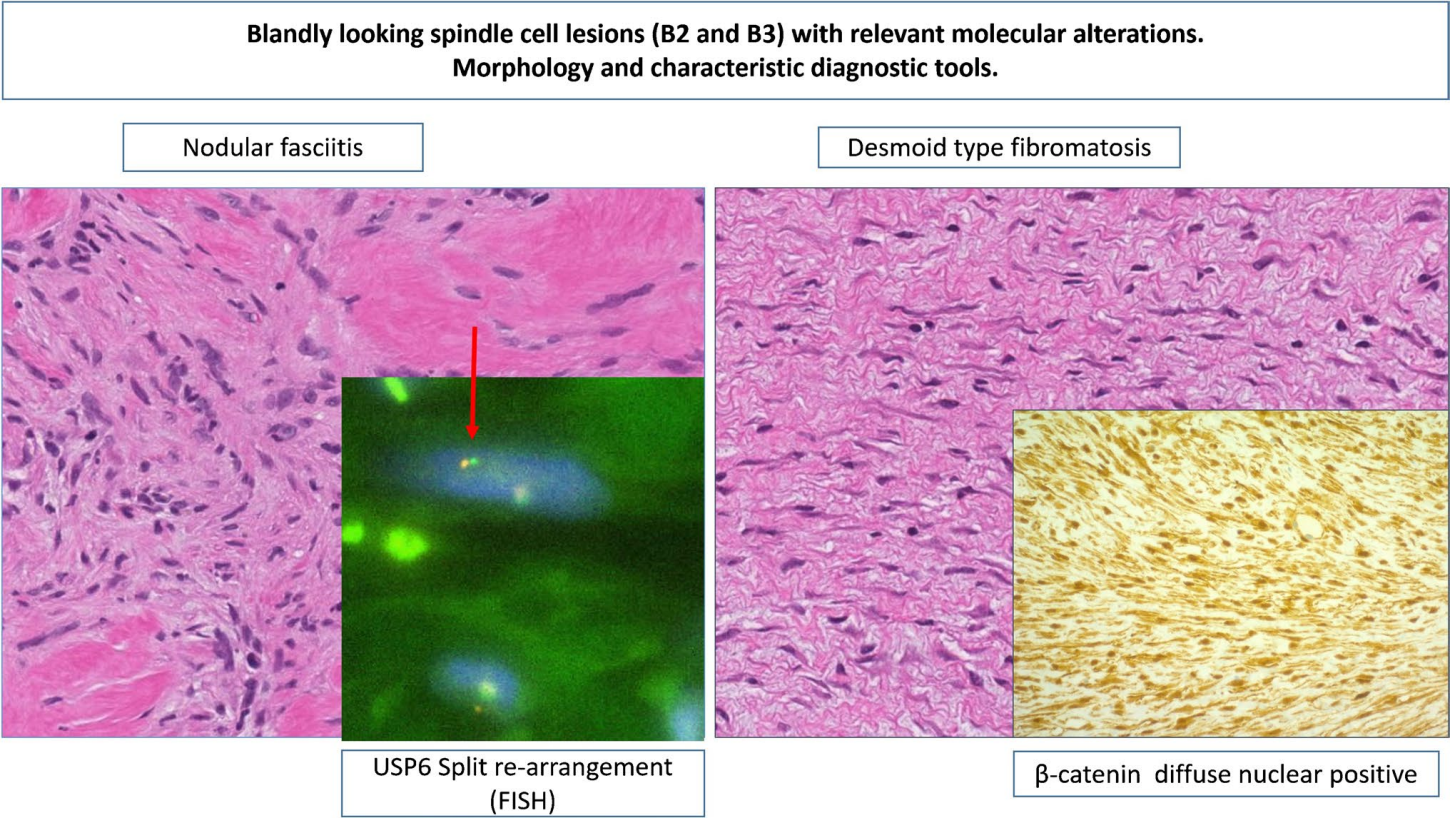
Blandly looking spindle cell lesions (B2 and B3) with relevant molecular alterations. Morphology and characteristic diagnostic tools. Nodular fasciitis with USP6 re-arrangement (with FISH). Desmoid type fibromatosis with diffuse nuclear β-catenin positivity

**Fig. 5:**
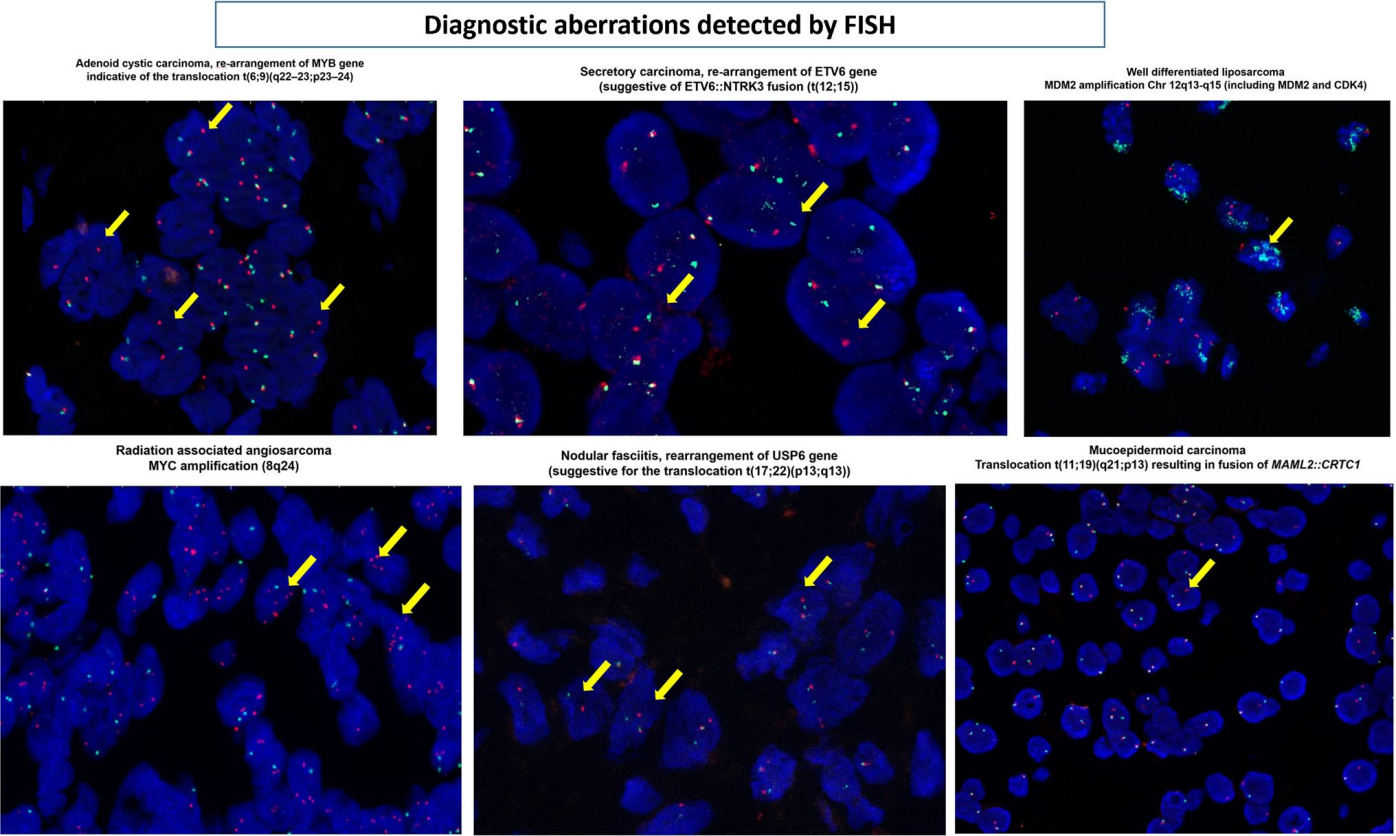
Illustration of diagnostic ISH (in these examples with FISH technology) assays, such as rearrangement of MYB gene, indicative of *MYB::NFIB* fusion t(6;9)(q22–23;p23–24 (AdCC), re-arrangement of ETV6 gene, suggestive of ETV6::NTRK3 fusion (secretory carcinoma), MAML2::CRTC1 translocation (mucoepidermoid carcinoma), MYC amplification (radiation-associated angiosarcoma), USP6 re-arrangement (nodular fascitis), and MDM2 amplification Chr 12q13-q15 (liposarcoma)

However, spindle cell lesions within the B3 category (desmoid-type fibromatosis) and some of the B2 category, such as nodular fasciitis, and myofibroblastic proliferations can benefit of additional molecular testing [1] (Table 3, Figs. 4 and 5).

**Table 3:** Spectrum of benign tumors (B2), tumors/lesions of uncertain malignant potential (B3), of vascular and lipomatous tumors. Characteristic diagnostic, immunohistochemical stain, and molecular test

Diagnosis	IHC panel	ISH assays	Molecular alterations via sequencing or NGS
Nodular fasciitis	**Strong expression of SMA and focal of desmin** Negative: CD34, cytokeratin, p63, CK5/6, nuclear β-catenin	**Rearrangement of USP6** gene (suggestive for the translocation t(17;22)(p13;q13))	No specific mutations
Desmoid type fibromatosis	**Expression of nuclear β-catenin** p63,SMA Negative: CD34, desmin, cytokeratin, CK5/6, ER, PR	Not relevant	**CTNNB1 mutations**
Myofbroblastoma	**Expression of desmin, CD34, ER, PR, AR** Negative: cytokeratin, S100, nuclear β-catenin, p63, CK5/6	**13q14 deletion**	No specific mutations
Hemangiomas Atypical vascular lesions Primary angiosarcoma	**Expression of CD31, CD34, ERG** **Ki67 (high in angiosarcoma)** **TN**	**Lack of MYC amplification (8q24)**	No specific mutations
Radiation-associated angiosarcoma	**Expression of CD31, CD34, ERG** **Ki67 (high)** **TN**	**Evidence of MYC amplification (8q24)**	No specific mutations
Lipoma Angiolipoma Well-differentiated liposarcoma (part of PT as heterologous component)	**Expression of S100** **Negative: MDM2**	**Lack of amplification of Chr 12q13-q15 (including MDM2 and CDK4)**	No specific mutations
Well-differentiated liposarcoma (not part of a PT)	**Expression of S100, MDM2**	**Evidence of amplification of Chr 12q13-q15 (including MDM2 and CDK4)**	No specific mutations
Myxoid liposarcoma	**Expression of S100**	**DDIT3 re-arrangement t(12,16)(q13;p11)**	***FUS::DDIT3*** ***EWSR1::DDIT3* t(12,22)(q13;p12)**

**Bold** indicates diagnostic entity specific tests. *ER*, estrogen; *PR*, progesterone; *AR*, androgen receptor; *TN*, triple negative

### **Nodular fasciitis**

This lesion is a self-limited blandly looking spindle cell proliferation mixed with inflammatory cells, with characteristic rapid clinical growth and a suggestive immunohistochemical phenotype (negative for most markers such as CD34, p63, CK5/6, cytokeratins, nuclear β-catenin, strongly positive for smooth-muscle actin (SMA) or focally for desmin) [1]. For confirmation, a specific FISH break apart probe for USP6 can be optionally performed if there is any doubt on the diagnosis, since a rearrangement of USP6 gene (suggestive for the translocation t(17;22)(p13;q13)) is present in over 85% of the cases [1, 37].

### **Desmoid-type fibromatosis**

This is a blandly looking spindle cell proliferation, resembling desmoid-type fibromatosis elsewhere in other organs and exhibiting a typical immunophenotype [1, 42]. On immunohistochemistry, there is a co-expression of p63, SMA, and nuclear β-catenin, while other markers such as cytokeratins, CD34, ER, PR, and Her2 are negative [1, 42]. In most instances, there is no need for further molecular testing, as the demonstration of aberrant nuclear expression of β-catenin along with a consistent expression of other immunohistochemical markers and the corresponding HE morphology is diagnostic on preoperative core biopsy samples. However, if there is still a diagnostic leak and/or the beta-catenin immunostain is not conclusive or uninterpretable, the possibility to evidence for the underlying CTNNB1 mutation through Sanger sequencing or an appropriate NGS panel are further options in selected cases [1, 42]. In such instances, the option for removal of the visible finding and aiming a definitive diagnosis on the surgical specimen should be followed.

### **Myofibroblastic proliferations**

Particular example of this group is the myofibroblastoma, which is composed of a circumscribed spindle cell proliferation with intervening collagenous stroma in between [1]. The HE morphology and the corresponding IHC expression profile (positivity for desmin, CD34, ER, PR, and AR) with lack of cytokeratins, EMA, S100, and nuclear beta-catenin) are diagnostic in most cases also and the diagnosis of myofibroblastoma can be rendered on preoperative core biopsy tissue [1, 36, 41]. In rare instance, an ISH probe with evidencing 13q14 deletion (in 70–80% of the cases) can be supportive, however not mandatory for this diagnosis.

## Molecular testing in vascular and lipomatous tumors

### **Vascular tumors**

Among vascular tumors diagnosed in the breast is the main differential diagnosis to differentiate between benign vascular tumors (such as the frequent capillary hemangioma or angiomatosis) and post-radiation-associated vascular lesions such as atypical vascular lesions or angiosarcoma vs primary angiosarcoma [1]. Additionally to the clinical picture (size of the lesion) and morphological criteria (nuclear atypia, growth pattern), further markers (both immunohistochemical and ISH probes) are often necessary to secure the definitive diagnosis. The presence of endothelial differentiation, demonstrated by positivity for CD31, CD34, ERG, or D2-40, and increased proliferative activity (high Ki67 index) within the endothelial cells support the diagnosis of a malignant vascular proliferation [1]. Diagnostic of post-radiation angiosarcoma is the evidence of MYC amplification (8q24) through FISH amplification probe, whereas in the absence of such amplification, depending on the clinical picture and nuclear morphology, size, and growth pattern, the diagnosis of an atypical vascular lesion or alternatively of primary angiosarcoma should be considered [1]. NGS-based sequencing can be applied as well if MYC status in ISH and/or the morphology remain equivocal [1].

### **Lipomatous tumors**

Lipomatous tumors in the breast pose a diagnostic challenge between benign lesions such as lipoma, angiolipoma, or atypical lipomatous tumor/well-differentiated liposarcoma (ALT/WDLS), the latter in pure form or in form of a heterologous element within a phyllodes tumor [1]. The morphological features (bland nuclear morphology and the lack of lipoblasts) together with characteristic immunohistochemical features (MDM2 and cytokeratin negativity along with S100 expression) support the diagnosis of a benign lipomatous proliferation. However, it should be kept in mind that MDM2 nuclear positivity may be found in histiocytes in the area of steatonecrosis. For this reason, the demonstration of amplification of Chr 12q13-q15 (including MDM2 and CDK4) via FISH is necessary for the diagnosis of ALT/WDLS [1]. An important point is that ALT/WDLS arising in patients with Li-Fraumeni syndrome are typically MDM2 negative; however, they show an overexpression of p53. Furthermore, it is of note that well-differentiated liposarcoma arising as heterologous elements within a phyllodes tumors lack MDM2 amplification, therefore in this context attributed to borderline PT and not to a malignant variant [1]. Another entity belonging to lipomatous tumors is myxoid liposarcoma, which typically exhibits DDIT3 rearrangement t(12,16)(q13;p11) as consequence of the *FUS::DDIT3* gene fusion or the *EWSR1::DDIT3* t(12,22)(q13;p12) gene fusion. DDIT3 rearrangements can be demonstrated through FISH break apart probes; however, for the detection of the fusion partner, a RNA-based NGS analysis may be required [1]. Pleomorphic liposarcoma often exhibit alterations in TP53 and RB1, which can be detected through NGS. However, these mutations, even though frequent, are not diagnostic for pleomorphic liposarcomas [1]. Thus, for the definitive diagnosis of pleomorphic liposarcoma, a consistent morphology (with demonstration of a pleomorphic sarcoma with presence of lipoblasts/lipoblastic differentiation) along with absence of MDM2 amplification (which would be rather consistent, if present, with dedifferentiated liposarcoma) is needed.

## Molecular testing as prognostic and predictive tool in breast cancer

The assessment of the predictive markers is mandatory in breast cancer diagnostics, as it allows tailoring specific adjuvant or neoadjuvant systemic therapies or targeted therapies in metastatic settings [10–13]. Established markers are hormone receptors (ER, PR), Her2 status, and proliferation index via Ki67 labelling, which must be determined in all newly diagnosed breast cancer and should be retested in recurring or metastatic lesions, if tissue availability is given [12, 43, 44]. Furthermore, the evidence of sequencing-based further alterations such as PIK3CA pathway, BRCA1/2 mutations, NTRK fusions, microsatellite instability (MSI), or mutations on ESR1 or ERBB2 genes are important tools to tailor targeted individual therapies [1, 10, 12, 39, 43–45]. 

## Hormone receptors (estrogen ER, progesteron PR)

Both ER and PR must be tested on each primary breast carcinoma and should be re-assessed on any recurring/metastatic lesions if tissue is available for testing, as differences in expression profile can occur in up to 50% of cases [10, 12, 43, 44]. Positive ER status is a pre-requisite for an endocrine therapy (e.g., aromatase inhibitors or selective estrogen receptor modulators) and is associated with a favorable prognosis [10–12, 44]. If PR is positive as well, these tumors, classified as luminal-A tumors, show a favorable outcome [10–12]. Established methodology is immunohistochemistry (according to internal and external quality assurance guidelines), which should provide the percentage of positively stained nuclei of the invasive tumor component (cut-off at least 1%, however, cases with positivity between 1 and 10% behave biologically similar to TNBC) [10–12]. Around 80% of BC are ER positive and up to 70% are PR positive.

## Her2 status including Her2 low and Her2 mutation status

Her2 status is another mandatory marker, which must be tested on each primary breast carcinoma and should be re-assessed on any recurring/metastatic lesions if tissue is available for testing. Differences in Her2 status are less frequent; however, also up to 30% immunohistochemical or ISH results can change during the disease course, possibly through clonal differentiation or clonal resistance to established therapies administered to the primary tumor [10–12, 43, 44, 46].

Routine Her2 testing methods are immunohistochemistry alone with complementary ISH probes or ISH probes for Her2 alone. Both approaches are approved by ASCO/CAP guidelines [10–12, 43, 44, 46]. A positive Her2 status, making the patient eligible for anti-Her2 therapy, requires an immunohistochemical score 3+ or an amplification of the ERBB2 gene in ISH irrespectively of the IHC result [44]. Approximately 10–15% of BC are Her2 positive [10, 44, 47, 48].

The newly described Her2-low category represents a subgroup of BC with immunohistochemical score 1+ or 2+ without amplification in ISH. Those cases are eligible for Trastuzumab-Derutexan (T-DXd) therapy in inoperable or metastatic breast carcinoma as second-line treatment [43, 45]. Her2 (ERBB2) activating point-mutations are often detected in ER-positive carcinomas undergoing sequencing through NGS, especially in metastatic invasive lobular carcinomas (in up to 8%). In these cases, a dual combination therapy regiments with anti-hormonal and anti-Her2 regiments with Neratinib can be discussed [8, 9, 43, 49].

## BRCA 1/2 mutations

BRCA 1/2 mutations via NGS sequencing became a routine tool according to the results of both Olympia studies to select patients eligible for PARP inhibitor therapies (such as olaparib, talazoparib) both in metastatic setting and in early breast cancer [43, 49, 50]. The Olympia studies provided evidence that germline BRCA 1/2 mutations are relevant in Her2-negative breast cancer both in triple-negative and hormone receptor-positive tumors as these subgroups have longer disease-free and metastasis-free survival after PARP inhibitor therapy [43, 49, 50]. In most cases, there is evidence for both somatic and germ line BRCA 1 /2 mutations in the given patients [43, 49, 50]. However, for therapeutic reasons, testing for BRCA 1/2 germline mutation is recommended (and performed from peripheral blood) [43, 49, 50]. On the other hand, the TBCRC-048 study showed a good clinical response to olaparib therapy in patients with metastatic breast cancer exhibiting somatic BRCA 1/2 mutations in their tumor tissue [51]. Therefore, it is advisable to include BRCA 1/2 genes in routinely applied NGS panels as well [43].

## PD-L1 status and immuncheckpoint inhibitors

Immuncheckpoint inhibitors (e.g., atezolizumab or pembrolizumab) are therapeutic options for metastatic TNBC and whose prerequisite is a positive PD-L1 status via immunohistochemical testing within the invasive tumor cells (TC) and/or in the accompanying immune cells (IC) [43, 52–54]. FDA-approved PD-L1 antibodies include the clones SP142 and 22C3 or SP263 immunohistochemical assays. The decision of which one must be performed varies in dependence of the trial validated and the checkpoint inhibitor that will be given to the patients and should thus be discussed with the treating oncologist. The assay therefore depends on the administered drug. Cutoffs for the decision in favor of treatment are least 1% of positive immune cells (determined with the clone SP142) for therapy with atezolizumab and a combined positivity score (CPS) of at least 10 (determined with the clone 22C3 or SP263) for the therapy with pembrolizumab [43, 52–54]. A recent meta-analysis suggests an association between high tumor mutational burden and longer overall survival in patients receiving immuncheckpoint inhibitor therapy [55].

## ESR1 mutations

Mutations in the ERS1 gene which codes ER occur in 20–40% of metastatic ER-positive luminal breast cancer and less often in early forms (1–2%) and can be detected via NGS sequencing [43, 49, 56, 57]. Endocrine resistance, which has been attributed to ESR1 mutations in ER-positive breast cancer, can be tailored by alternative combination therapy with aromatase inhibitors and CDK4/6 inhibitors [43, 49, 56–58]. For the detection of ESR1 mutation, it is probably more adequate to perform the test in liquid biopsy if available than on paraffin embedded tissues, which was endorsed by recent clinical guidelines as well [10].

## NRTK pathway

NTRK activation and consecutive fusion of the three NTRK transmembrane proteins through translocations is a rare event in breast cancer (<1%); however, in secretory carcinomas, NTRK fusion is more often detected (up to 50%). Anti-Pan NTRK therapy in TNRK IHC-positive BC cases has been shown to have good therapy response [43, 49, 59, 60]. In case of positive NRTK protein expression, a NRTK translocation has to be confirmed via ISH probes or preferentially via RNA-based NGS sequencing [43, 49, 59, 60].

## PIK3CA pathway

Activating and other type of mutations in the PIK3CA pathway can be detected via NGS or Sanger sequencing in up to 40% of hormone receptor-positive breast cancer and is considered todays a both prognostic and predictive factor [43, 49, 61, 62]. Mutation in Exons 9 and 20 is pre-requisite to administer PIK3CA-inhibitor combination therapy (alpelisib and fulvestran) in ER-positive metastatic breast cancer [10, 43, 49]. Mutations, or allelic losses in further PI3K pathway-associated genes such as AKT1 and PTEN, provide additional therapy options [10, 43, 49]. Loss of PTEN occurs in up to 40%, often in TNBC and is associated with poor prognosis, while mutations in AKT1 (< 10% in ER-positive BC) result in increased proliferative activity through mTOR-pathway inhibition [10, 43, 49]. Testing for PIK3CA mutations is usually performed on tumor tissue from the latest progression if available rather than on liquid biopsy [10, 43, 49].

## Mismatch repair proteins

Microsatellite instability (MSI), which can be assessed by immunohistochemical analysis of the four conventional mismatch repair (MMR) proteins (MLH1, PMS2, MSH2, MSH6), PCR amplification followed by capillary electrophoresis or by NGS, is a rare phenomenon at breast cancer (<1% detection rate), which can be considered for check-point inhibitor (Pembrolizumab) therapy in metastatic breast cancer [10, 49, 63]. However, based on current data, no causal biological interaction exists between the presence of tumor infiltrating lymphocytes and deficient mismatch repair protein expression [49]. The two antibody procedure (PMS2 and MSH6 testing) instead of reflex testing all four mismatch repair proteins can be used as a reliable substitute to identify MMR deficiency [64]. Reflex testing of all four MMR proteins as suggested by Aiyer et al. should be performed in tumors with unclear results in the two-step algorithm [64].

## Multigene expression tests and Ki67 index

Multigene expression tests have become part of standard diagnostic tests in ER-positive Her2-negative early breast cancer, in nodal-negative and nodal-positive patients providing risk scores to tailor the decision between adjuvant endocrine therapy alone or in combination with chemotherapy [10–12, 49, 65, 66]. Multigene expression tests are applied especially in those instances where conventional clinicopathological parameters including also the Ki67 labelling index do not allow the decision in favor of or against adjuvant chemotherapy [10, 12, 66, 67]. The currently approved five tests (Oncotype DX, Mammaprint, Endopredict, Prosigna, and Breast Cancer Index (BCI)) per dato are all run on paraffin embedded tumor tissue via NGS or qRT-PCR methodology [10, 66, 68]. These multigene expression tests have been validated in prospective (MINDACT for Mammaprint, TailorX and NSAB B20 for Oncotype DX) or in retrospective trials/cohorts (ABCSG 6/8 for Endopredict, ATAC and ABCSG 8 for Prosigna) [10, 66, 68]. Oncotype DX, Mammaprint, and the BCI assays are carried out in a reference central laboratory, whilst Endopredict and Prosigna can be tested de-centralized in the individual pathology laboratory [10, 66, 68]. The clinical need for risk scores via multigene expression tests is high especially within the “grey zones of all clinico-pathological parameters” which does not allow a clear therapy decision (especially in Ki67 index grey zone defined as < 5–30% >) [10, 66, 68].

## Metastatic breast cancer

The possibility to administer targeted therapies in the metastatic setting has been regulated by several clinical guidelines, such as ESMO ESCAT or AGO [10, 69, 70]. The proposed workflow by ESMO ESCAT recommends (1) a biopsy of the metastatic lesion and (2) a retesting of established biomarkers such as ER/PR and Her2 on the biopsied metastatic lesion [69, 70]. In luminal type Her2-negative metastases, PIK3CA mutational status should follow, in triple-negative tumors PD-L1 status via IHC and germ-line BRCA mutations should be completed [69, 70]. Optional assessments include MSI, TMB or NTRK, ESR1 mutations, Her2 low status, or somatic BRCA mutation testing [69, 70].

## Declarations

Not applicable as the paper is an overview review article and no own research data are presented.
